# Cell wall targeted *in planta* iron accumulation enhances biomass conversion and seed iron concentration in Arabidopsis and rice

**DOI:** 10.1111/pbi.12557

**Published:** 2016-04-07

**Authors:** Haibing Yang, Hui Wei, Guojie Ma, Mauricio S. Antunes, Stefan Vogt, Joseph Cox, Xiao Zhang, Xiping Liu, Lintao Bu, S. Charlotte Gleber, Nicholas C. Carpita, Lee Makowski, Michael E. Himmel, Melvin P. Tucker, Maureen C. McCann, Angus S. Murphy, Wendy A. Peer

**Affiliations:** ^1^ Center for Direct Catalytic Conversion Of Biomass to Biofuels (C3Bio) Purdue University West Lafayette IN USA; ^2^ Department of Horticulture Purdue University West Lafayette IN USA; ^3^ Department of Biological Sciences Purdue University West Lafayette IN USA; ^4^ Biosciences Center National Renewable Energy Laboratory Golden CO USA; ^5^ X‐ray Science Division Advanced Photon Source Argonne National Laboratory Argonne IL USA; ^6^ National Bioenergy Center National Renewable Energy Laboratory Golden CO USA; ^7^ Department of Botany and Plant Pathology Purdue University West Lafayette IN USA; ^8^ Department of Bioengineering Northeastern University Boston MA USA; ^9^ Department of Chemistry and Chemical Biology Northeastern University Boston MA USA; ^10^ Department of Plant Science and Landscape Architecture University of Maryland College Park MD USA; ^11^ Department of Environmental Science and Technology University of Maryland College Park MD USA; ^12^ Present address: Department of Biology Colorado State University Fort Collins CO 80523 USA

**Keywords:** biofuel, cell wall, secretion, iron‐binding peptide, carbohydrate‐binding module, iron concentration

## Abstract

Conversion of nongrain biomass into liquid fuel is a sustainable approach to energy demands as global population increases. Previously, we showed that iron can act as a catalyst to enhance the degradation of lignocellulosic biomass for biofuel production. However, direct addition of iron catalysts to biomass pretreatment is diffusion‐limited, would increase the cost and complexity of biorefinery unit operations and may have deleterious environmental impacts. Here, we show a new strategy for *in planta* accumulation of iron throughout the volume of the cell wall where iron acts as a catalyst in the deconstruction of lignocellulosic biomass. We engineered CBM‐IBP fusion polypeptides composed of a carbohydrate‐binding module family 11 (CBM11) and an iron‐binding peptide (IBP) for secretion into Arabidopsis and rice cell walls. *CBM‐IBP* transformed Arabidopsis and rice plants show significant increases in iron accumulation and biomass conversion compared to respective controls. Further, *CBM‐IBP* rice shows a 35% increase in seed iron concentration and a 40% increase in seed yield in greenhouse experiments. *CBM‐IBP* rice potentially could be used to address iron deficiency, the most common and widespread nutritional disorder according to the World Health Organization.

## Introduction

As the world population continues to increase and fossil resources are finite, alternative feedstocks for fuels and other chemicals are being developed to meet this growing demand (Hill *et al*., 2006). Nongrain lignocellulosic biomass feedstocks, such as rice straw, are sustainable resources because they are renewable, abundant and are not used as human food (Himmel *et al*., 2007). In addition to fuel, low‐cost lignocellulosic biomass can be converted into high‐value products in selective biorefinery processes. Unfortunately, thermal and chemical conversions require high‐energy inputs and the efficiency of biochemical conversion is limited by biomass recalcitrance. A strategy to reduce initial energy inputs and facilitate the deconstruction of cell walls (Wei *et al*., 2011) involves use of transition metal catalysts, such as Fe^2+^. However, pretreatments in which the metal catalysts are directly added to biomass will increase unit operations and costs, and the high concentrations of metal required to penetrate the cell walls present potential environmental problems of water usage and disposal.

Furthermore, a method that increases iron accumulation in rice grain is important to combat iron deficiency in human populations which rely on this low‐iron grain for subsistence (Meng *et al*., 2005; Goto *et al*., 1999). Transition metal uptake, transport and homeostatic mechanisms are tightly coordinated, and efforts to engineer iron‐enriched crops for food and metal hyperaccumulating plants for phytoremediation have achieved limited success after 25 years of research (Palmer and Guerinot, 2009). Translation of Ni^2+^ hyperaccumulation mechanisms to Fe^2+^, Cu^2+^ and Mn^2+^ accumulation is limited as cytosolic Fe^2+^, Cu^2+^ and Mn^2+^ levels required for effective cell wall catalysis are toxic to living plants. Therefore, the challenge is to avoid cellular toxicity and, at the same time, accumulate transition metals throughout the volume of the cell wall, both to reduce biomass recalcitrance in vegetative tissues and to increase iron concentration in grain. Here, a combination of strategies was used to tailor plants for increased transition metal concentration to facilitate direct catalysis of cell wall deconstruction. An unexpected consequence of this approach was enhancement of iron in rice grains.

## Results and discussion

### Design and synthesis of CBM‐IBP constructs

**Figure 1:**
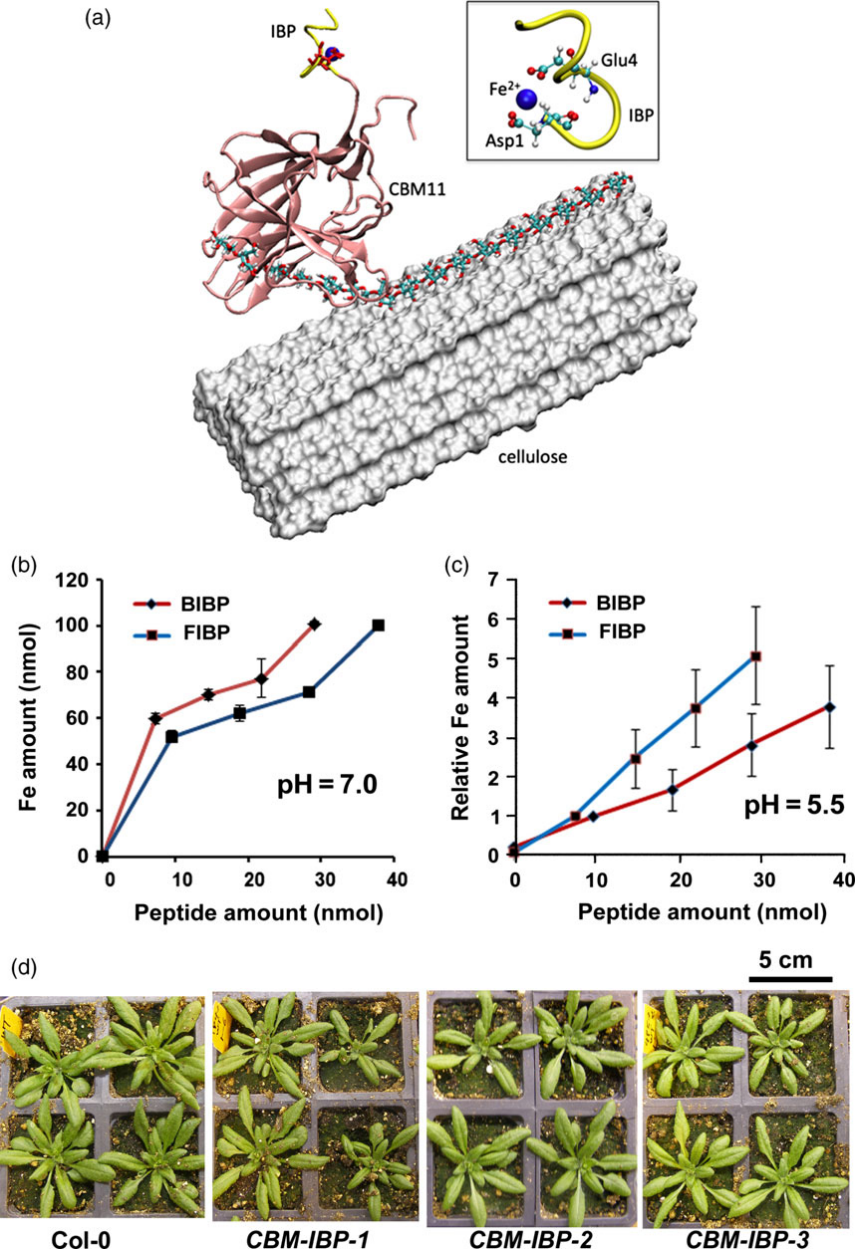
Design and generation of *CBM‐IBP* constructs and Arabidopsis plants. (a) Putative model of CBM11‐IBP (*Ct* CBM11: pink, synthetic blood IBP: yellow) binding to the cell wall. The CBM11‐IBP is placed on the hydrophobic surface of a microfibril, represented as white surface. An edge chain was pulled up from the cellulose surface and bound in the binding cleft of *Ct* CBM11. Inset: The Fe^2+^‐binding site in IBP is highlighted (red: oxygen atoms; Fe^2+^: blue sphere). (b, c) Iron‐binding assays of the synthetic blood iron‐binding peptide (BIBP) and ferritin iron‐binding peptide (FIBP) at pH 7.0 and pH 5.5. Values are mean ± standard deviation (SD), *n* = 3 technical replicates. (d) Representative images of 28‐days Col‐0 and three independent homozygous *CBM‐IBP* Arabidopsis transgenic lines; their growth was comparable to Col‐0.

Figure 1a shows the approach used to increase transition metal concentration *in planta* for efficient cell wall deconstruction by fusing an iron‐binding peptide (IBP), a carbohydrate‐binding module (CBM) to bring the IBP near carbohydrate bonds in the cell wall and a signal peptide (SP) so that the SP‐CBM‐IBP polypeptide would be secreted to the cell wall. Several constitutive and inducible constructs were designed that contained the SP‐CBM‐IBP polypeptide and different tags, for example haemagglutinin (HA) or a fluorescent protein (Figure 1a and Figure S1a). Two iron‐binding peptides (IBP) were synthesized and analysed for metal binding: a minimal ferritin‐binding peptide based on the ferritin monomer and a synthetic iron‐binding motif based on the blood iron‐binding peptide that we had previously isolated and found that it bound iron across a wide range of pH. Both the synthetic blood IBP DLGEQYFKG (Lee and Song, 2009) and the ferritin IBP LAEEKREGYER (Lawson *et al*., 1991) bound iron *in vitro* at cytosolic pH ~7.0 and apoplastic pH ~5.5 (Figure 1b,c). An extensin secretion signal peptide (SP) (De Loose *et al*., 1991) was chosen for secretion of the SP‐CBM‐IBP polypeptide into the cell wall. Signal peptides have been used to successfully secrete carbohydrate‐binding modules (CBMs) in poplar, Arabidopsis and rice (Cho and Cosgrove, 2000; Choi *et al*., 2003).

*Clostridium thermocellum* CBMs were used to target the IBPs to carbohydrate components of the cell wall. The pool of CBMs is diverse, with each CBM targeting different polysaccharides in the cell wall (Boraston *et al*., 2004; Shoseyov *et al*., 2006). Five CBMs with an SP N‐terminal fusion and a C‐terminal pH‐insensitive fluorescent protein mCherry (Shaner *et al*., 2005) were expressed in Arabidopsis under control of a constitutive promoter (SP‐CBM‐mCherry) and analysed for secretion (Figure S1b). Of the five, three showed intracellular fluorescent signals, and CBM11, which binds primarily 1,4‐ß and 1,3‐1,4‐ß mixed linked glucans, and CBM22, which binds primarily 1,3‐ß and 1,4‐ß glucans (Hashimoto, 2006), showed signals localized to the cell wall, indicating that these two CBMs were successfully secreted, although some intracellular signal was observed (Figure S1b). Higher fluorescent signals were observed in the *SP‐CBM11‐mCherry* plants compared to *SP‐CBM22‐mCherry* plants (Figure S1b); therefore, CBM11 was used for further analyses. IBPs were then fused to the secreted CBM11 (SP‐CBM11‐IBP) to target the iron to carbohydrate bonds in the cell wall as shown in Figure 1a. Modelling of SP‐CBM11‐IBP docking to cellulose microfibrils suggests that iron targeting to cell walls is possible (Wu *et al*., 2013), although this remains to be demonstrated *in planta*. CBM11 fused to the minimal ferritin iron‐binding peptide (SP‐CBM11‐FIBP) did not show obvious signals in the cell wall in *Arabidopsis thaliana* (data not shown) and was not analysed further. Secreted CBM11 fused to the synthetic blood iron‐binding peptide and HA (SP‐CBM11‐IBP‐HA), hereafter called CBM‐IBP, showed a range of relative expression levels (Figure S1c). Whereas metal hyperaccumulating plants often show reduced growth (Palmer and Guerinot, 2009), a wide range of *CBM‐IBP* expression levels had no detrimental effect on plant growth when expressed in *Arabidopsis thaliana* (Figure 1d and Figure S1d).

### CBM‐IBP localizes to cell walls in Arabidopsis

**Figure 2:**
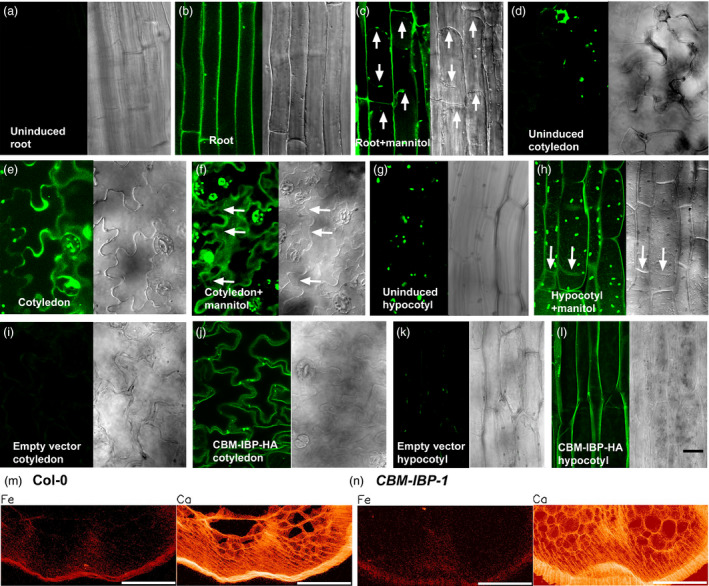
CBM‐IBP and iron ions localize to cell walls in Arabidopsis. (a*‐*h) Expression and localization of the oestrogen‐inducible CBM‐IBP‐Dendra2 fluorescent protein in Arabidopsis without (a, d, g) and with (b, c, e, f, h) 4 μm 4‐hydroxytamoxifen for 16–40 h. (c, f, h) Cell wall and intracellular signals were observed after plasmolysis with 0.6 m mannitol. Arrows indicate the position of the plasma membrane following plasmolysis. (i**–**l) Immunohistochemistry of CBM‐IBP‐HA in Arabidopsis with anti‐HA showed cell wall and intracellular localization in hypocotyls and cotyledons; controls transformed with empty vector plasmid showed no signal. Scale bar = 20 μm. (m, n) Iron localization analysed by X‐ray fluorescence microscopy showed cell wall localization of Fe in inflorescence stem sections of 4‐week‐old Col‐0 and *CBM‐IBP‐1* plants (see methods). Ca^2+^ localization is shown as a reference to indicate cell walls. Scale bar = 100 μm.

To determine whether CBM‐IBP was secreted to the cell wall, CBM‐IBP localization was analysed under the control of a tamoxifen‐inducible promoter (CBM‐IBP‐Dendra2) or a constitutive promoter (CBM‐IBP‐HA). No autofluorescence in roots and only chlorophyll autofluorescence in hypocotyls and cotyledons were observed in uninduced plants (Figure 2a,d,g). After induction, CBM‐IBP‐Dendra2 signals were observed in roots (Figure 2b,c), cotyledons (Figure 2e,f) and hypocotyls (Figure 2h), and apoplastic localization was observed following plasmolysis (Figure 2c,f,h). Upon induction, the intracellular localization was most pronounced in younger tissue, that is cells close to the root tip (Figure 2c), compared to mature tissue, consistent with the greater activity of the secretory system in actively growing cells (De Loose *et al*., 1991; Hall and Cannon, 2002; Lamport *et al*., 2011); unsecreted CBM‐IBP may bind to the cell wall during senescence. Immunohistochemistry of constitutively expressed CBM‐IBP‐HA also showed apoplastic localization (Figure 2j,l), and no signal was detected in plants transformed with the empty vector (Figure 2i,k). Further, X‐ray fluorescence microscopy, in which each pixel is analysed for elemental fluorescence, showed that iron signals were localized to the cell walls of the epidermis as well as the vascular tissue in mature stems (Figure 2m,n); calcium (Figure 2m,n) and zinc (not shown) signals were observed in the cell walls throughout the stem. Iron quantification using an absolute scale could not be obtained due to variations in section thickness. These data suggest that CBM‐IBP could bind the iron at the cell wall and suggested the possibility of iron deposition in the cell wall may have occurred via the secretory pathway. Whereas both inducible and constitutive expression resulted in CBM‐IBP accumulation at the cell wall, plants with constitutively expressed HA fusion polypeptides were chosen for further analyses due to tamoxifen toxicity.

### *CBM‐IBP* enhances iron concentration in Arabidopsis

**Figure 3:**
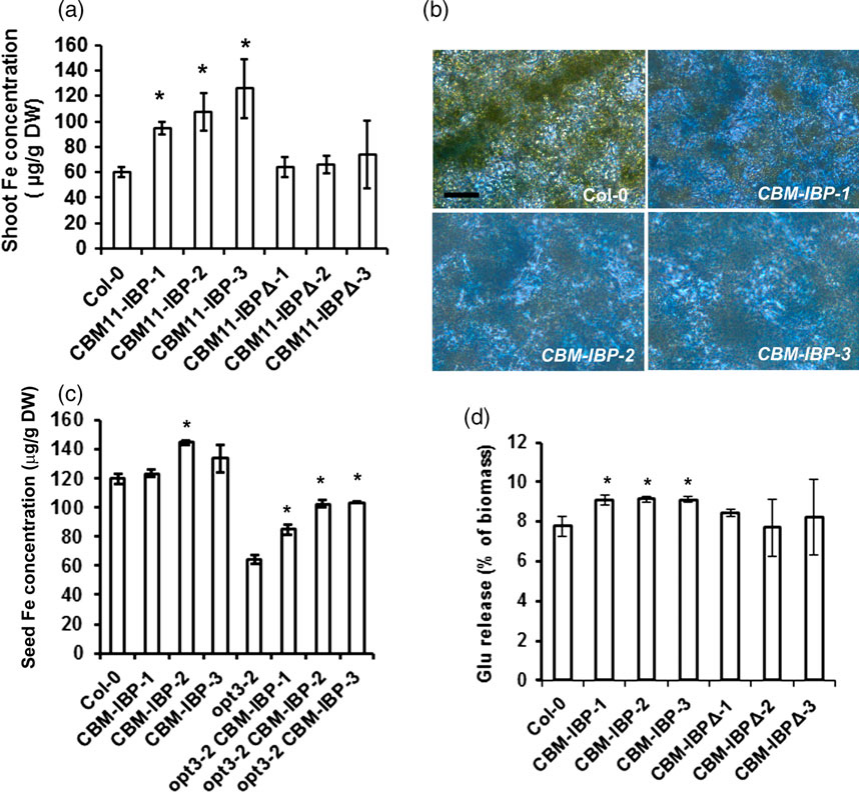
*CBM‐IBP* enhances iron concentration and biomass conversion in Arabidopsis. (a) Shoot iron concentration was greater in *CBM‐IBP* Arabidopsis lines compared to Col‐0 and *CBM‐IBPΔ* harbouring mutations in iron‐binding sites. (b) Perls' staining showed more iron concentration (blue) in *CBM‐IBP* Arabidopsis leaves than Col‐0 leaves. Scale bar = 50 μm. (c) Iron concentration in seeds was increased by expression of *CBM‐IBP* in Arabidopsis mutant *opt3‐2, oligopeptide transporter 3‐2*, and Arabidopsis mutant has less iron in seeds than wild type. Values are mean ± SD,* n* = 3 biological replicates. *,* P *<* *0.05, Student's *t*‐test, compared to corresponding control. (d) Glucose (Glu) yield from *CBM‐IBP* Arabidopsis dry shoots was greater than Col‐0 and *CBM‐IBPΔ*. Values are mean ± SD,* n* = 3 biological replicates. *, *P *<* *0.05, by Student's *t*‐test compared to corresponding control.

*Arabidopsis thaliana* plants expressing *CBM‐IBP* showed increased iron concentration in shoots and seeds and enhanced biomass conversion in shoots (Figure 3). *CBM‐IBP* shoots contained 60%–70% more iron compared to wild‐type control plants (*P *<* *0.05, Figure 3a). Perls' Prussian blue staining was used to visualize iron accumulation directly *in planta*. *CBM‐IBP* Arabidopsis leaves showed more staining than controls, indicating increased iron accumulation (Figure 3b). To determine whether the iron‐binding peptide was responsible for the increased iron concentration, the DLGEQYFKG residues in CBM‐IBP were mutated to VLGVIYIVG (*CBM‐IBPΔ*) which abolishes the iron‐binding motif E/DXXE (Fang and Wang, 2002; Lawson *et al*., 1991). *CBM‐IBP∆* Arabidopsis lines with a range of expression levels comparable to the *CBM‐IBP* lines were analysed (Figure S2a and Figure S1c). *CBM‐IBPΔ* plants had iron concentrations comparable to wild type (Figure 3a and Figure S2b); therefore, the CBM‐IBP, but not CBM‐IBPΔ, binds iron and increases iron amounts *in planta*.

The iron accumulation in seeds was analysed to assess whether CBM‐IBP could also enhance iron accumulation in other tissues. Iron is naturally high in Arabidopsis seeds, and *CBM‐IBP‐2* seeds showed increased iron accumulation compared to wild type (*P *=* *0.002), and *CBM‐IBP‐1* and *CBM‐IBP*‐*3* were similar to wild type (*P *=* *0.09, *P *=* *0.11, respectively; Figure 3c). However, significantly more iron accumulated in seeds when *CBM‐IBP* was expressed in the *oligopeptide transporter 3‐2* (*opt3‐2*) mutant background (*P *<* *0.01, Figure 3c) which has reduced iron concentration in seeds (Stacey *et al*., 2008). These results indicate that CBM‐IBP‐mediated iron increases are distinct from OPT3‐dependent iron accumulation in Arabidopsis seeds (Stacey *et al*., 2008), and indicate that CBM‐IBP can increase iron accumulation in plants that have low amounts of iron.

### CBM‐IBP enhances biomass conversion in Arabidopsis

Previously, we developed a dilute acid/Fe^2+^ pretreatment for biomass that increased the yield of sugar monomers (Wei *et al*., 2011). Moreover, the hot water/Fe^2+^ and hot water/Fe^3+^ pretreatments were also effective in releasing more simple sugars than hot water alone pretreatment (Kamireddy *et al*., 2013; Liu *et al*., 2009; Zhao *et al*., 2011). Arabidopsis plants expressing *CBM‐IBP* or *CBM‐IBPΔ* were analysed using this process, that is without adding exogenous Fe^2+^. *CBM‐IBP* shoots yielded 20% more glucose and 15% more xylose compared to controls (*P *<* *0.05, Figure 3d and Figure S2c). The enhanced release of sugar monomers can be attributed to iron binding by IBP, as *CBM‐IBPΔ* shoots showed glucose and xylose release comparable to wild type (*P *>* *0.05, Figure 3d and Figure S2c). Total cellulose and ethanol‐soluble and ethanol‐insoluble sugars in *CBM‐IBP* biomass were not different from wild type and *CBM‐IBP∆* controls (*P *>* *0.05, Figure S2d–f). Therefore, a functional iron‐binding motif in CBM‐IBP is necessary to enhance cell wall hydrolysis in *CBM‐IBP* plants. This is also consistent with iron reduction in biomass recalcitrance under acidic conditions in pretreatments (Wei *et al*., 2011) and demonstrates that iron accumulated *in planta* appears to function in the same manner.

### CBM‐IBP enhances iron concentration and biomass conversion in rice

**Figure 4:**
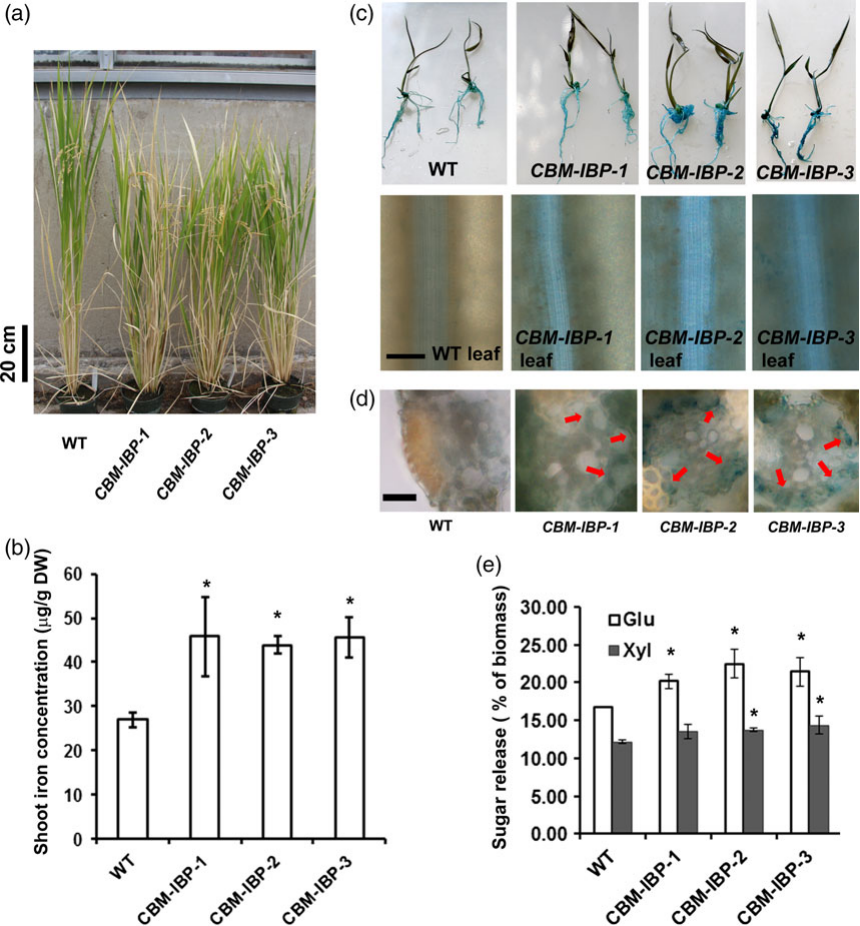
CBM‐IBP enhances iron concentration and biomass conversion in rice. (a) Representative image of 120‐d‐old wild‐type (WT) and independent homozygous *CBM‐IBP* transgenic lines. (b) Shoot iron concentration was greater in *CBM‐IBP* rice lines than WT. (c) Perls' staining showed greater iron concentration in *CBM‐IBP* rice leaves than WT leaves. Scale bar = 100 μm. (d) Perl's staining on cross sections shows iron localization in vascular tissue. Scale bar = 20 μm. (e) Glucose and xylose yields from *CBM‐IBP* rice straw were greater than that from WT. Values are mean ± SD,* n* = 3 biological replicates. *, *P *<* *0.05, by Student's *t*‐test compared to corresponding control.

There is some question whether the success of iron accumulation in increasing monomer sugar release in Arabidopsis would transfer to grasses. Arabidopsis cell walls are typical of dicotyledonous species, but the cell walls of grass species such as rice contain additional noncellulosic polysaccharides. Further, Arabidopsis uses a reduction strategy for iron uptake, while rice uses a combination of reduction and chelation (Palmer and Guerinot, 2009). Wild‐type rice was transformed with *CBM‐IBP* to test whether the same *in planta* technology can be translated to crop rice. Homozygous transgenic rice lines that expressed *CBM‐IBP* were selected for analyses (Figure 4a and Figure S3a). *CBM‐IBP* rice shoots had ~60% increase in iron accumulation compared to wild type (*P *<* *0.05, Figure 4b), similar to the increase observed in *CBM‐IBP* Arabidopsis. Perls' Prussian blue staining also showed increased iron levels in *CBM‐IBP* rice roots and leaves (Figure 4c), with staining most pronounced in xylem cells (Figure 4d). Without exogenous Fe^2+^ in the pretreatment, release of glucose and xylose from *CBM‐IBP‐2* rice shoots was comparable to *CBM‐IBP* Arabidopsis plants, yielding 25% more glucose and 15% more xylose compared to controls (*P *<* *0.05, Figure 4e). Total cellulose, ethanol‐soluble and ethanol‐insoluble sugars in *CBM‐IBP* rice biomass were similar to wild type (*P *>* *0.05, Figure S3b,c,d). Further, the amount of glucose released was greater in the 2M trifluoroacetic acid (TFA)‐soluble fraction and reduced in the nonsoluble pellet (*P *<* *0.05, Figure 3c). As rice and Arabidopsis have similar cellulose structures and differential effects of CBM11 expression in rice and Arabidopsis are unlikely, *CBM‐IBP∆* analyses were not repeated in rice. These results demonstrate that iron accumulated *in planta* efficiently facilitates biomass deconstruction, although the effects of iron on the synthesis of other polymers to enhance saccharification cannot be ruled out.

### CBM‐IBP increases yield and iron concentration in rice seeds

**Figure 5:**
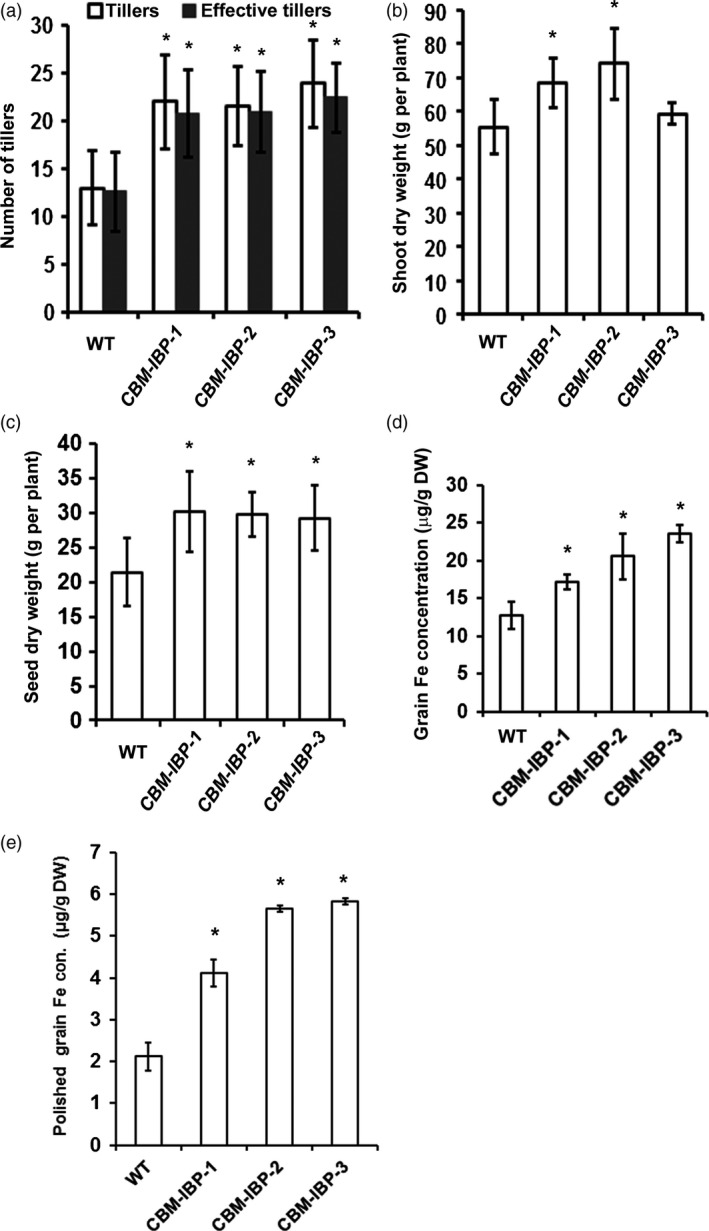
*CBM‐IBP* rice plants showed increased yield and grain iron concentration. (a‐c) Number of tillers (effective tillers, tillers producing seeds), shoot dry weight and seed dry weight were greater in homozygous *CBM‐IBP* rice transgenic lines compared to wild type (WT). Values are mean ± SD,* n* = 8 biological replicates. (d, e) Iron concentration was greater in *CBM‐IBP* dehusked brown rice and polished white grains than controls. Values are mean ± SD,* n* = 3 biological replicates. *, *P *<* *0.05, Student's *t*‐test, compared to corresponding control.

As iron hyperaccumulation may reduce growth and/or yield, the growth of *CBM‐IBP* rice plants was analysed in detail. Rice expressing *CBM‐IBP* senesced slightly earlier than wild type based on the colour and chlorophyll levels of older flag leaves when the panicles were fully filled (Figure 4a; Figure S3f). The *CBM‐IBP* rice homozygous lines had reduced height compared to wild type (Figure 4a and Figure S3e). Whereas short stature is a favourable trait for grain production in rice, it is not for biomass production. However, *CBM‐IBP* rice plants developed more tillers (*P *<* *0.05, Figure 5a), and, offsetting their short statures, the shoot dry weight of *CBM‐IBP‐1* and *CBM‐IBP‐2* rice was greater than wild type (*P *<* *0.05, Figure 5b). Plants in the greenhouse were grown at the same density as in the field, and tillering of wild‐type rice in the greenhouse was comparable to field‐grown rice. *CBM‐IBP* rice plants also produced ~40% more seeds per plant compared to wild type (*P *<* *0.05, Figure 5c), which will be re‐evaluated in field trials. Chlorophyll quantifications were greater in *CBM‐IPB* mature green flag leaves and less in yellow flag leaves compared to wild type (*P *<* *0.05, Figure S3f). The increased chlorophyll concentration in the *CBM‐IBP* flag leaves is consistent with increased seed yield and iron concentrations in biomass and seeds (described below). Rice tillering is modulated by strigolactones, and DWARF 27 (D27), an iron‐binding isomerase that converts *trans*‐β‐carotene into 9‐*cis*‐β‐carotene in the strigolactone biosynthetic pathway (Alder *et al*., 2012; Lin *et al*., 2009). Increased iron may affect D27 activity and/or function and subsequently influence tillering activity, although how iron may affect D27 or other factors regulating tillering is unknown. Furthermore, iron promotes chlorophyll biosynthesis (Chereskin and Castelfranco *et al*., 1982) which may have increased the biomass yield in transgenic plants; however, such hypotheses need to be tested in future experiments.

The iron accumulation in *CBM‐IBP* dehusked brown rice seeds (brown rice, also known as unmilled or unpolished rice) was analysed to determine whether an iron increase similar to *CBM‐IBP* Arabidopsis occurred. *CBM‐IBP* rice seeds had 35% (*CBM‐IBP‐1*) to 37% (*CBM‐IBP‐3*) more iron than wild‐type seeds (*P *<* *0.05, Figure 5d; Table S1). *CBM‐IBP* rice contained 17–23 μg iron/g dry weight, which is comparable to iron accumulation in rice overexpressing soya bean ferritin (12–20 μg iron/g dry weight, Qu *et al*., 2005) or hydroponically grown *NFP* brown rice overexpressing two transgenes, nicotianamine and ferritin in the endosperm (17 μg iron/g dry weight, Wirth *et al*., 2009). Iron levels in milled rice (white rice or polished rice) increased ~two‐fold in *CBM‐IBP‐1* and ~three‐fold in *CBM‐IBP‐2* and *CBM‐IBP‐3* compared to wild type (*P *<* *0.05, Figure 5e). The iron increase in *CBM‐IBP* white rice (4–6 μg iron/g dry weight) was comparable to *NFP* white rice (~7 μg iron/g dry weight). The average seed weight and size (length, width, area) were similar among *CBM‐IBP* and wild‐type rice grains (*P *>* *0.1, Figure S4a–e), and therefore, the average iron amount per grain was greater in *CBM‐IBP* than wild type. Furthermore, peptide‐chelated iron may be more bioavailable for absorption (Lee and Song, 2009). Analysis of the ion profile in both Arabidopsis and rice seeds indicated some increases in other metal ions for which there are toxicity concerns, such as Cd, Co, Cr and Cu (Table S1). However, the concentration of the metal ions measured was below the toxic limits set by FDA (www.fda.org) based on consumption of 1 kg dry brown rice per day. For reference, the average consumption is ~0.5–0.6 kg rice per day by individuals in the highest rice‐consuming countries. If implemented in the field, cultivar selection for biofuels and/or biofortification characteristics should be considered because of genetic variation in mineral concentration among rice cultivars (2–45 μg iron/g dry weight brown rice, Zheng *et al*., 2011).

Here, a triple fusion polypeptide was used successfully to accumulate iron in the cell wall while avoiding cellular toxicity: (i) the signal peptide from a native cell wall extension protein to provide efficient secretion (De Loose *et al*., 1991), (ii) a synthetic blood iron‐binding peptide to efficiently bind iron and (iii) CBM11 to direct the IBP‐bound iron to cell wall polysaccharides to prevent re‐uptake of free iron into the cell (Palmer and Guerinot, 2009) resulting in enhanced biomass conversion. In contrast to the millimolar iron added to feedstock postharvest (Wei *et al*., 2011), *CBM‐IBP* plants contain micromolar iron that efficiently and effectively increases saccharification yield. These results indicate that *in planta* iron targeting to the cell wall can effectively increase saccharification yields in biochemical conversion processes. The release of simple sugars in *CBM‐IBP* rice was at least comparable to the pretreatment of corn stover in Wei *et al*. (2011), suggesting that the plant engineering approach is more efficient than the conventional iron loading approach. The current cultural practices of growing rice, which includes foliar iron applications, would not need to be altered to realize increased iron concentration in *CBM‐IBP* rice. Further, it appears that *CBM‐IBP* rice more efficiently utilizes the iron in the foliar spray than wild type based on the increased amounts of chlorophyll and iron in leaves. It is important to note that biomass recalcitrance varies among different plant tissues, for example leaves versus stems and apical versus basal stems. Some of the observed differences in pretreatment and enzymatic digestibility could be attributed to differences in *CBM‐IBP* plant growth compared to the controls (i.e. shorter stature, thicker stems, more tillers).

It would be unrealistic to consider that straw from *CBM‐IBP* rice would be the only part of the plant used, especially with the need to feed a growing global population. An unintended consequence of expressing *CBM‐IBP* was a ~40% increase in seed yield and 35% increase in seed iron accumulation. Given that a 3% increase in yield is considered significant (Baldwin *et al*., 2012), we will determine whether the enhanced traits are observed under field conditions. The increased iron accumulation in brown and white rice could address iron deficiency, with an apparently low potential for toxicity from other metals. Allergens from the peptides would also be of concern. The IBP has no predicted antigenic determinants, and CBM11 has five; for comparison, rice α‐expansin (CBMs are homologous to expansins) has seven (http://imed.med.ucm.es). Taken together, this technology could be used for both enhanced conversion of rice straw biomass to biofuel and increased iron‐enriched grain yield to support both global energy and food demands.

## Experimental procedures

### Constructing the binding model of CtCBM11‐IBP to cell wall

The atomistic model of the *Ct*CBM11 (*Clostridium thermocellum* CBM11) was constructed using the reported NMR structure 2LR0 (Viegas *et al*., 2013). The blood iron‐binding peptide was initially generated as a α‐helix and attached to the C‐terminal end of *Ct*CBM11. The parameters of the Fe^2+^‐binding site in ferritin monomer (Lawson *et al*., 1991), for example the distances between Fe^2+^ and oxygen atoms, were used to restrain the binding site in the blood Fe‐binding peptide. An energy minimization of *Ct*CBM11‐IBP system was then conducted for 2000 steps of steepest decent minimization followed by 2000 steps adopted basis Newton–Raphson (ABNR) minimization using CHARMM (Brooks *et al*., 2009). A previously equilibrated cellulose Iβ microfibril (Beckham *et al*., 2012; Payne *et al*., 2011; Wu *et al*., 2013) was used to construct the *Ct*CBM11‐IBP/cellulose complex. The microfibril was composed of four layers, which consist of 3, 4, 5 and 6 cellulose chains from the top layer to the bottom layer, respectively. Each cellulose chain has 16 glucose residues. As *Ct*CBM11 is characterized as a type B subfamily of CBMs and binds to a single cellodextrin chain, the *Ct*CBM11 was placed on the hydrophobic surface with a single ‘edge’ cellodextrin chain bound to its binding cleft. The CHARMM22 force field parameters (Brooks *et al*., 2009; MacKerell *et al*., 1998) were used for the peptide and the C35 carbohydrate force field parameters (Guvench *et al*., 2008, 2009) were used for the cellulose during energy minimization. Fe^2+^ and two oxygen atoms at the Fe^2+^‐binding site were harmonically restrained. A final minimization of the entire system was run for 1000 steps of steepest decent minimization followed by 1000 steps ABNR minimization.

### Iron‐binding peptides and assays

The peptides DLGEQYFKG (ferritin iron‐binding peptide, FIBP, molecular weight 1057) and LAEEKREGYER (blood iron‐binding peptide, BIBP, MW 1380) were synthesized by Cambridge Research Biochemicals (www.crbdiscovery.com). For IBP‐binding assay at pH 7.0 phosphate buffer, free Fe^2+^ forms insoluble compound with phosphate and removed by centrifugation. IBP‐bound Fe in the supernatant was determined by orthophenanthroline which results a red solution when binds with Fe^2+^. The iron‐binding assay at pH 7.0 was performed in 500 μL binding solution [20 mm MES pH 7.0; 0.5 mm FeCl_2_; peptide (2 mg/mL, 50 mm phosphate buffer pH 7.0): 0, 5, 10, 15, and 20 μL; H_2_O up to 500 μL, 2 mm final phosphate] at room temperature for 1 h with gentle shaking according to Lee and Song (2009). The binding solution was spun at 3500 × g for 30 min to remove precipitates. The iron concentration in the supernatant was determined using a colorimetric method (adapted from Harris, 1998) by orthophenanthroline which results in a red solution when binds with Fe^2+^. Hydroquinone is added to prevent oxidation of Fe^2+^ to Fe^3+^. One hundred μL supernatant was transferred to a new microcentrifuge tube to which 20 μL 1% hydroquinone and 50 μL 0.25% orthophenanthroline and 830 μL H_2_O were added to make the final volume 1 mL, and the absorbance at 510 nm was determined. The iron‐binding assay at pH 5.5 was performed as above with the following modifications due to the solubility of the iron at pH 5.5: 100 μL binding solution (20 mm MES pH 5.5; 2.5 mm FeCl_2_) was used for the assays and 20 μL supernatant was blotted onto polyvinylidene difluoride (PVDF) membrane. The membranes were washed with 20 mm MES pH 5.5 buffer twice for 5 min each time. The membranes were developed in 5 mL developing solution (100 μL 1% hydroquinone, 250 μL 0.25% orthophenanthroline) and imaged, and the colour intensity was analysed with ImageJ software (rsbweb.nih.gov/ij/).

### Constructs

All primers used in this work are listed in Table S2. The extensin signal peptide (SP) and the carbohydrate‐binding module (CBM) cassettes *SP‐CBMs‐mCherry* and *SP‐CBM11‐Roo* were constructed using overlapping PCR primers. After digestion with BamHI and SacI, the *SP‐CBMs‐mCherry* and *SP‐CBM11‐Roo* cassettes were cloned into *pCAMBIA2300* (www.cambia.org/daisy/cambia/585). The *SP‐CBM11‐IBP* (blood iron‐binding peptide) cassette called *CBM‐IBP* was amplified from *pCambia2300‐SP‐CBM11‐Roo* using the primers CaccSP‐5′ and BIBP‐3′ and cloned into Gateway entry vector *pENTR/TOPO* (Invitrogen, www.invitrogen.com). The *CBM‐IBPΔ* cassette containing the mutated blood iron‐binding motif (*BIBPΔ*) was amplified from *pENTR‐CBM‐IBP* using the primers CaccSP‐5′ and BIBPΔ‐3′ and cloned into pENTR/TOPO. The constructs were analysed by PCR and restriction digests, and were sequenced using the M13 forward primer. The plasmids *pENTR‐CBM11‐IBP/IBPΔ* were cut with MluI, and the *CBM11‐IBP/IBPΔ* cassettes were transferred to *pGWB14* (Nakagawa *et al*., 2007) (with C‐terminal HA fusion) and/or the oestrogen‐inducible vector *pIST04* (modified from *pEarleyGate101* with N‐terminal oestrogen‐inducible elements: *FMV:NEV* + 10x *NIP* promoter and C‐terminal Dendra2 fusion) (Jásik *et al*., 2013) using LR reactions (Invitrogen).

### Plant transformation

Arabidopsis was transformed with the constructs by the floral dip method (Clough and Bent, 1998). The resulting transformants were selected on 0.45% phytagel plates containing quarter‐strength Murashige and Skoog (MS) basal salts, pH 5.5, at 22 °C, 14 h 100 μmol/m^2^/s supplemented with kanamycin (50 mg/L), hygromycin (50 mg/L) or phosphinothricin (6 mg/L, for Bar resistance), and genotyped by PCR. T1 heterozygous and T2 or T3 homozygous *CBM11‐IBP/IBPΔ* Arabidopsis plants were selected and analysed with kanamycin and hygromycin and PCR genotyping. *CBM11‐IBP‐Dendra2* Arabidopsis plants were selected with phosphinothricin and BASTA spray. Rice was transformed with *CBM11‐IBP* and *CBM11‐Roo* using Agrobacterium‐mediated method described previously (Nishimura *et al*., 2006). Briefly, seeds from *O. sativa* var. *japonica* ‘Nipponbare’ were surface‐sterilized and germinated on N6 medium [Chu's N6 medium with vitamins supplemented with 2 mg/L 2,4‐dichlorophenoxyacetic acid (2,4‐D), 0.1 g myo‐inositol, 0.3 g/L casein hydrolysate, 2.878 g l‐proline and 3% sucrose, pH, 5.8, solidified with 4 g phytagel] to initiate calli. After incubation for 3–4 weeks at 30 °C, light (110 μmol/m^2^/s), actively growing calli (yellowish white) were collected and proliferated on the same medium to be used for *Agrobacterium* cocultivation (3 days, at 28 °C in dark). Infected calli were washed with sterile water and selected with N6 medium with 50 mg/L hygromycin and 250 mg/L carbenicillin for 3–4 weeks at 30 °C, light at 110 μmol/m^2^/s. Hygromycin‐resistant calli were transfer to rice shoot regeneration medium (4.4 g/L MS salts, 2.0 mg/L 6‐benzylaminopurine, 30 g/L sucrose, 7 g/L TC agar), and regenerated shoots were transferred to full‐strength MS medium with 2% sucrose and 0.7% agar. Twenty transgenic lines for Arabidopsis and more than ten lines for rice were generated. Three lines were continued to homozygosity for detailed analyses. T3 homozygous transgenic plants were selected with hygromycin (50 mg/L) and confirmed by PCR genotyping.

### Plant growth conditions

Arabidopsis wild‐type and *CBM11‐FIBP*/*CBM‐IBP* seedlings were grown as above except as indicated for specific treatments. Arabidopsis wild‐type and *CBM11‐FIBP/CBM‐IBP* plants were grown in the greenhouse in soil under natural light conditions, except the day length was extended to 16 h with HID lights (150 μmol/m^2^/s) in the winter. See www.hort.purdue.edu/hort/facilities/greenhouse/hlaTech.shtml for more information. Plants were watered with 1/10 strength Hoagland's nutrient solution (Barac *et al*., 2004) supplemented with 100 μm iron every 2 weeks or with 1/10 strength Hoagland's nutrient solution supplemented with a 0.5 mm iron solution (Sequestrene 330 Fe, Becker Underwood, INC, Ames, IA) foliar spray once every 2 weeks (3 times in total). The floral spray was developed as a more environmentally friendly iron application method for biofuel feedstock production. Rice wild‐type and *CBM‐IBP* seedlings were grown on 0.45% phytagel plates, containing quarter‐strength MS, pH 5.5, 22 °C, 14 h light (100 μmol/m^2^/s) except as indicated for specific treatments. Rice wild‐type and *CBM‐IBP* plants were grown in the greenhouse in calcined clay (Profile Greens calcined clay granules in 9‐cm pots) with drip irrigation under semi‐dry and natural light conditions, and in the winter, the day length was extended to 16 h with HID lights (250 μmol/m^2^/s). The rice plants were watered and fertilized with 1/10 Hoagland's solution supplemented with 50 μm iron or 1/10 Hoagland's solution supplemented with a 0.5 mm iron foliar spray every 2 weeks after flowering (three applications in total). Before harvest, tiller numbers were counted and plant heights were measured from the root–shoot junction to the tip of the tallest flag leaf. Plant biomass and seeds were harvested and dried in a laminar flow hood at room temperature for 1 week, and dry weights were recorded. The *CBM‐IBP* Arabidopsis and rice plants contained similar amounts of iron in both soil and foliar Fe application, so the iron concentration was independent of the fertilization method.

### Quantitative RT‐PCR analysis of transgenic Arabidopsis and rice

Total RNA was isolated from 7‐days Arabidopsis or 10‐days rice seedlings using the RNeasy Mini Kit according to the vendor's manual (Qiagen, www.qiagen.com). RNA was quantified and treated with TURBO DNA‐free^™^ DNase (Ambion RNA by Life Technologies, www.invitrogen.com). First‐strand cDNA was synthesized from three μg of total RNA with the SuperScript^®^ III reverse transcription kit followed by reverse transcription, according to the vendor's manual (Invitrogen). Quantitative RT‐PCR was performed using the CFX96 real‐time PCR detection system (Bio‐Rad, www.Bio-Rad.com) and GoTaq^®^ qPCR Master Mix (Promega, www.promega.com). See Table S2 for the primers.

### Iron staining

Arabidopsis and rice seedlings were vacuum‐infiltrated with Perls' Prussian blue stain solution (equal volumes of 4% [v/v] HCl and 4% [w/v] K‐ferrocyanide) for 15 min. The plant samples were incubated for 30 min, rinsed three times with water and stored in 70% ethanol. The staining was imaged using a Zeiss Observer Z1 microscope (Carl Zeiss, Jena, Germany). Stained samples were imbedded in Neg‐50 frozen section medium (Richard‐Allan Scientific, Kalamazoo, MI). 100‐μm‐thick sections were prepared in Microm HM550 (Microm International GmbH, Walldorf, Germany) and imaged using a Zeiss Observer Z1 microscope (Carl Zeiss, Jena, Germany).

### Immunohistochemical localization, immunofluorescence and fluorescent protein localization studies

Arabidopsis 5‐d seedlings were prepared for immunolocalization as described in Peer *et al*. (Peer *et al*., 2009) Briefly, seedlings were fixed in 4% *p*‐formaldehyde in microtubule‐stabilizing buffer (MTSB) solution (50 mm PIPES, 5 mm EGTA, and 5 mm MgSO4) and 5% DMSO for 1 h. They were washed with MTSB + 0.1% Nonidet P‐40 for 10 min (five times). The seedlings were permeabilized for 1 h in 10% DMSO and 1% Nonidet P‐40 and then blocked with 3% BSA in MTSB for 3 h. The seedlings were incubated overnight (37 °C) with anti‐HA (1 : 250, Santa Cruz Biotechnology, www.scbt.com), in 3% BSA in MTSB. They were then washed for 10 min (six times) in 0.1% Triton X‐100, for 10 min (three times) in MSTB, and then incubated for 3 h at 37 °C in goat anti‐rabbit‐Alexa Fluor 488 (1 : 250) in 3% BSA/MTSB. The seedlings were washed in MTSB/0.1% Triton X‐100 and then in water for 10 min each (five times). Immunofluorescence analysis was performed using a Zeiss LSM 710 confocal laser scanning microscope (Carl Zeiss, Jena, Germany). The following settings were used: for Alexa Fluor 488: 488 nm argon laser at 3.5%, pinhole 90 μm, emission 493–630 nm, gain 650, pixel dwell 6.3 μs; for YFP: 514 nm argon laser at 13%, pinhole 90 μm, emission 519–621 nm, gain 833, pixel dwell time 3.15 μs; and for mCherry: 543 nm argon laser at 60%, pinhole 45 μm, emission 578–696 nm, gain 821, pixel dwell time 6.3 μs. The oestrogen‐inducible *CBM‐IBP‐Dendra2* Arabidopsis plants were germinated on ¼ strength MS plates. Three‐day‐old seedlings were transferred to plates with ¼ strength MS containing 4 μm 4‐hydroxy tamoxifen for 16–40 h. CBM‐IBP‐Dendra2 signals were analysed using 488 nm argon laser at 24%, pinhole 90 μm, emission 493–577 nm, gain 820, pixel dwell 3.15 μs.

### X‐ray fluorescence microscopy

Four‐week‐old bolted Arabidopsis plants were sprayed with iron solution (0.5 mm) once. Inflorescence stems were dissected and imbedded in 5% agar when cooled to ~50 °C. 100‐μm‐thick sections were prepared using a TPI Vibratome 1000. Sections were sent to Argonne National Laboratory for analysis according to method described in Inouye *et al*. (2014).

### Elemental analyses

Arabidopsis and rice shoot tissues were harvested when fully senescent, air‐dried and shaken to remove the seeds. Dry *Arabidopsis* and rice shoots were ground twice to pass through a 20‐mesh (1‐mm) screen using a Wiley Mill. The husk was removed from dry rice seeds to obtain brown rice; brown rice was polished to whited rice according to Wirth *et al*. (2009). Both brown and white rice grains were ground into a fine powder using a mortar and pestle. An aliquot of the ground samples was used to measure the metal concentrations with modifications to published procedures (Stacey *et al*., 2008; Vansuyt *et al*., 2007; Wei and Layzell, 2006). Briefly, 20 mg of dry, ground sample was digested in 0.4 mL 25% (v/v) nitric acid (Trace Metal Grade, Fisher Scientific), at 70 °C, overnight. After digestion, the extract was diluted to 5 mL with fresh Millipore (Synergy Water Purification System) de‐ionized H_2_O (the final nitric acid concentration was 2%) and analysed quantitatively for iron and other metal ions using inductively coupled plasma‐optical emission spectroscopy (ICP‐OES) by the Chemical Analysis Laboratory at the University of Georgia. Trial experiments indicated that the recovery rates for the ions during the nitric acid extractions were between 97% and 102%; thus, the original data have been presented without adjustments.

### Biomass pretreatment and conversion to sugars

Arabidopsis and rice shoot tissues were harvested when fully senescent, air‐dried and shaken to remove the seeds, ground twice to pass through a 20‐mesh (1‐mm) screen using a Wiley Mill and then tested for total sugar release through hot water pretreatment combined with enzymatic hydrolysis, using a high‐throughput method. The advantage of using a high‐throughput (HTP) type pretreatment procedure is that it can process large numbers of biomass samples with small sample sizes to test sugar release (Decker *et al*., 2009; Selig *et al*., 2010; Studer *et al*., 2010, 2011). Briefly, 5 mg of ground biomass from each plant line was weighed manually in sample replicates into individual wells on 96‐well Hastelloy plates; water was added, the plates sealed, clamped and subjected to hot water pretreatment and cosaccharification, using a high‐throughput analytical process developed by NREL. The hot water pretreatment was conducted at 180 °C for 17.5 min. Following hot water pretreatment, the seals were pierced with a 96‐point punch to allow the addition of buffer and enzymes. The pH of pretreated biomass slurry was buffered at pH 5.0 by addition of 1 m sodium citrate buffer containing the desired enzymes (as described below) to ensure an optimal condition for enzymatic saccharification. A total volume of 40 μL of buffer and enzyme was added to each well; the enzyme used was Novozymes CTec2 (3 mg enzyme/g biomass). The 96‐well plate was resealed, and the enzymatic cosaccharification was carried out at 40 °C for 70 h. After the cosaccharification, the sugar release was analysed using glucose oxidase/peroxidase (GOPOD) assay for glucose and xylose dehydrogenase (XDH) assay for xylose as described previously (Gao *et al*., 2013; Selig *et al*., 2010).

### Rice grain size analyses

100‐grain rice seeds were collected from each plant and dry weights determined on a balance. Scanned pictures of collected rice grains were analysed using SmartGrain software program (Tanabata *et al*., 2012).

### Cellulose and sugar quantifications

Arabidopsis and rice shoot tissues were harvested when fully senescent, air‐dried and shaken to remove the seeds, ground twice to pass through a 20‐mesh (1‐mm) screen using a Wiley Mill. To determine cellulose concentration of rice straw and Arabidopsis shoot biomass, 15 mg samples were weighed into conical glass centrifuge tubes with screw caps, three biological replicates each. After adding 3 mL of a mixture of acetic acid/water/nitric acid (8/2/1, v/v/v), the suspension was incubated at 100 °C for 90 min with occasional mixing (Updegraff, 1969). After cooling, the tubes were centrifuged at 2500 ***g*** for 3 min and the supernatant discarded. The pellet was washed three times with water, centrifuged at 2500 g for 3 min and water discarded each time. The pellet was resuspended in 3 mL water and 250 μL was taken for phenol–sulphuric assay (Dubois *et al*., 1956); 250 μL of 5% phenol was added to each tube and mixed; and 2.5 mL of H_2_SO_4_ was added and vortexed to mix. Samples were incubated overnight at ambient temperature and read at 500 nm compared to cellulose standards (Sigmacell; Sigma).

To remove soluble sugars, 0.5 g dry biomass was incubated with 10 mL 50% ethanol at 70 °C for 20 min. The supernatant was dried under evaporator and sugar content was determined by phenol–sulphuric acid assay (Dubois *et al*., 1956). The pellet was washed once with 50% ethanol and five times with water. Alcohol‐insoluble residues were freeze‐dried and used for neutral sugar analysis.

Neutral sugar composition was analysed according to Gibeaut and Carpita (1991). Briefly, wall material (alcohol‐insoluble residues) was hydrolysed in 2 N trifluoroacetic acid (TFA) containing 0.5 μmol of myo‐inositol (internal standard) for 90 min at 120 °C. The TFA was evaporated in a stream of N_2_. Sugars were dissolved in 100 μL of 1 m NH_4_OH and 0.5 mL of 20 mg/mL NaBH_4_ in DMSO (w/v) and incubated at 45 °C for 90 min with occasional vortex mixing. The solution was neutralized with 100 μL of glacial acetic acid, and then, 100 μL of 1‐methylimidazole (Sigma) followed by 0.75 mL of acetic anhydride was added, and the mixture was incubated at 42 °C for 30 min. Water (1.5 mL) was added to destroy unreacted acetic anhydride, and when cool, the alditol acetates were partitioned into CH_2_Cl_2_. The entire reaction was completed in 1‐dram vials sealed with Teflon‐lined caps. Derivatives were separated by gas‐liquid chromatography (GLC) on a 0.25 mm × 30 m column of SP‐2330 (Supelco, Bellefonte, PA). Temperature was held at 80°C during injection, then ramped to 170 °C at 25 °C/min and then to 240 °C at 5 °C/min, with a 10‐min hold at the upper temperature. Helium flow was 1 mL/min with split‐less injection. The electron impact mass spectrometry (EIMS) was performed with a Hewlett‐Packard MSD at 70 eV and a source temperature of 250 °C.

### Antigenicity analyses

Antigen analyses were conducted using the precision tool at the Immunomedicine Group at University of Madrid (http://imed.med.ucm.es/Tools/antigenic.pl). The synthetic iron‐binding peptidase based on the blood iron‐binding sequence DLGEQYFKG (Lee and Song, 2009), CtCBM11 2LRP_A from the crystal structure and rice alpha‐expansin AAL24493.1. The prediction tool is based on the algorithms in Kolaskar and Tongaonkar (1990).

## Supporting information


**Figure S1** Expression of *CBM‐mCherry* and *CBM‐IBP* in Arabidopsis.
**Figure S2** Cellulose and sugar analysis of Col‐0, *CBM‐IBP* and *CBM‐IBPΔ* biomass.
**Figure S3** Characterization of *CBM‐IBP* rice plants: expression levels, plant height, chlorophyll concentration, and cellulose and sugar composition.
**Figure S4** Characterization of wild type (WT) and *CBM‐IBP* rice grains.
**Table S1** Seed element profile of *CBM‐IBP* and control Arabidopsis and rice plants (μg/g dry weight).
**Table S2** Primer list.
