# Supplementary material for: Cell wall targeted in planta iron accumulation enhances biomass conversion and seed iron concentration in Arabidopsis and rice
Source: Plant Biotechnol J. 2016 Apr 7;14(10):1998–2009. doi: 10.1111/pbi.12557 (PMC5043494; doi:10.1111/pbi.12557)
Supplement: Supplementary file 1 — Figure S1 Expression of CBM‐mCherry and CBM‐IBP in Arabidopsis. Figure S2 Cellulose and sugar analysis of Col‐0, CBM‐IBP and CBM‐IBPΔ biomass. Figure S3 Characterization of CBM‐IBP rice plants: expression levels, plant height, chlorophyll concentration, and cellulose and sugar composition. Figure S4 Characterization of wild type (WT) and CBM‐IBP rice grains. Table S1 Seed element profile of CBM‐IBP and control Arabidopsis and rice plants (μg/g dry weight). Table S2 Primer list. [file PBI-14-1998-s001.docx]

**Supplementary data**

**Cell wall targeted *in planta* iron accumulation enhances biomass conversion and seed iron concentration in Arabidopsis and rice**

Haibing Yang^1,2,3^, Hui Wei^4^, Guojie Ma^1,2^, Mauricio S. Antunes^1,3*^, Stefan Vogt^5^, Joseph Cox^1,2^, Xiao Zhang^2^, Xiping Liu^1,2^, Lintao Bu^6^, S. Charlotte Gleber^5^, Nicholas C. Carpita^3,7^, Lee Makowski^8,9^, Michael E. Himmel^4^, Melvin P. Tucker^5^, Maureen C. McCann^1,3^, Angus S. Murphy^1,2,10,^†, Wendy A. Peer^1,2,10,11^

^1^Center for Direct Catalytic Conversion Of Biomass to Biofuels (C3Bio), Purdue University, West Lafayette, IN 47907, USA

^2^Department of Horticulture, Purdue University, West Lafayette, IN 47907, USA

^3^Department of Biological Sciences, Purdue University, West Lafayette, IN 47907, USA

^4^Biosciences Center, National Renewable Energy Laboratory, Golden, CO 80401, USA

^56^X-ray Science Division, Advanced Photon Source, Argonne National Laboratory, Argonne, IL 60439

^6^National Bioenergy Center, National Renewable Energy Laboratory, Golden, Colorado

^7^Department of Botany and Plant Pathology, Purdue University, West Lafayette, Indiana 47907-2054

^8^Department of Bioengineering, Northeastern University, Boston, Massachusetts 02115

^9^Department of Chemistry and Chemical Biology, Northeastern University, Boston, Massachusetts 02115

^10^Department of Plant Science and Landscape Architecture, University of Maryland, College Park, MD 20742, USA

^11^Department of Environmental Science and Technology, University of Maryland, College Park, MD 20742, USA

*Current address: Department of Biology, Colorado State University, Fort Collins, CO 80523, USA

†Corresponding author

**Supplementary Table 1.** Elemental profile of CBM-IBP and control Arabidopsis and rice seeds (µg g^-1^ dry weight)

| Element | Col-0 | CBM-IBP-1 | *opt3-2* | *opt3-2*  CBM-IBP-1 | WT  (rice) | CBM-IBP-1 (rice) | CBM-IBP-2 (rice) |
| --- | --- | --- | --- | --- | --- | --- | --- |
| Al | 9.2 ± 0.29 | 8 ± 0.12* | 7.7 ± 1.71 | 6.2 ± 0.44 | 6.7 ± 1.68 | 6.3 ± 2.53 | 7.4 ± 1.44 |
| B | 18.7 ± 0.59 | 18.9 ± 0.66 | 12.8 ± 0.65 | 13.4 ± 0.51 | 1.4 ± 0.21 | 2.0 ± 0.08* | 2.79 ± 0.61* |
| Ba | 3.18 ± 0.17 | 3.19 ± 0.46 | 1.4 ± 0.45 | 1.9 ± 0.20 | 0.27 ± 0.07 | 0.14 ± 0.01 | 0.28 ± 0.11 |
| Ca | 7687 ± 568 | 8454 ± 434 | 4946 ± 347 | 5780 ± 222* | 160 ± 10.6 | 183 ± 6.91* | 178 ± 10.30* |
| Cd | 3.3 ± 0.05 | 3.12 ± 0.05 | 2.8 ± 0.20 | 2.8 ± 0.2 | 0.96 ± 0.03 | 1.16 ± 0.05* | 1.05 ± 0.07* |
| Co | 1.32 ± 0.05 | 1.09 ± 0.03* | 0.8 ± 0.01 | 0.6 ± 0.01* | 0.14 ± 0.02 | 0.26 ± 0.03* | 0.33 ± 0.07* |
| Cr | 2.11 ± 0.07 | 2.02 ± 0.02 | 1.7 ± 0.04 | 1.6 ± 0.05* | 0.44 ± 0.08 | 0.67 ± 0.07* | 0.82 ± 0.08* |
| Cu | 23.7 ± 0.7 | 21.97 ± 1.46 | 21.7 ± 2.44 | 11.4 ± 1.80* | 4.09 ± 1.07 | 4.6 ± 0.40 | 7.9 ± 2.88* |
| Fe | 119.5 ± 3.2 | 123.2 ± 2.27 | 64.3 ± 2.92 | 84.7 ± 3.37* | 12.7 ± 1.82 | 17.1 ± 0.93* | 17.5 ± 2.63* |
| K | 8071 ± 704 | 7846 ± 1611 | 6531 ± 390 | 5488 ± 674 | 2652 ± 238 | 3291 ± 183 | 5810 ± 1092 |
| Mg | 5476 ± 111 | 5364 ± 234 | 4445 ± 108 | 4289 ± 149 | 992 ± 28.82 | 1160 ± 100.82 | 1019 ± 25.12 |
| Mn | 53.6 ± 0.99 | 58.96 ± 5.72 | 49.84 ± 1.38 | 44.6 ± 3.41 | 17.8 ± 2.06 | 19.98 ± 2.35* | 20.7 ± 2.07* |
| Mo | 4.5 ± 0.08 | 4.2 ± 0.09* | 3.8 ± 0.05 | 3.7 ± 0.12 | 1.7 ± 0.23 | 1.97 ± 0.08 | 2.0 ± 0.16 |
| Na | 195.8 ± 5.71 | 220.9 ± 89.2 | 230.8 ± 23.51 | 153.4 ± 48.61 | 27.1 ± 5.03 | 28.8 ± 2.95 | 66.0 ± 24.76 |
| Ni | 2.45 ± 0.13 | 2.01 ± 0.09* | 1.5 ± 0.03 | 1.2 ± 0.05* | 0.61 ± 0.13 | 0.73 ± 0.08 | 0.97 ± 0.09 |
| P | 9847 ± 228 | 9618 ± 185 | 897 3± 157 | 9050 ± 290 | 2487 ± 122 | 3262 ± 484 | 3232 ± 264 |
| Pb | 8.4 ± 0.4 | 8.08 ± 0.05 | 6.9 ± 0.38 | 6.3 ± 0.32 | 1.79 ± 0.23 | 2.8 ± 0.19* | 3.25 ± 0.11* |
| Si | 145.2 ± 14.6 | 140.1 ± 8.9 | 69.0 ± 3.65 | 62.5 ± 1.53 | 67.5 ± 19.58 | 58.4 ± 11.09 | 92.4 ± 3.80 |
| Sr | 16.1 ± 1.16 | 17.8 ± 1.05 | 11.3 ± 0.96 | 11.8 ± 0.45 | 0.77 ± 0.17 | 0.66 ± 0.07 | 0.63 ± 0.11 |
| Zn | 72.5 ± 0.78 | 70.9 ± 1.82 | 65.0 ± 1.48 | 64.8 ± 4.95 | 24.2 ± 3.05 | 28.4 ± 0.14 | 20.6 ± 2.59 |

Values are mean ± SD, n = 3 biological replicates. *, *P* ≤ 0.05, Student’s *t*-test, compared to corresponding control. A different set of *CBM-IBP-2* seeds were used for iron determination in Figure 5d. There is no statistical difference in iron between the values shown in Supplementary Table 1 and Figure 5d.

**Supplementary Table 2. Primer list**

| Primer name | Primer sequence |
| --- | --- |
| ssmCherryF2/BamHI | ACG GAT CCA TGG GAA AAA TGG CTT CTC TAT TTG CCA CAT TTT TAG TGG TTT TAG T |
| ssmCherryF2/BamHI | ACG GAT CCA TGG GAA AAA TGG CTT CTC TAT TTG CCA CAT TTT TAG TGG TTT TAG T |
| mCherry_CBM11/overR | CAC CGA CAG CCA ATC CAA GAC CCA GCT CGT CAT GAG ATC TCT TGT A |
| mCherry_CBM11/overF | TGA CGA GCT GGG TCT TGG ATT GGC TGT CGG TGA AAA AAT GCT GG |
| CBM11R/SacI | GTG AGC TCT CAA GCA CCA ATC AGC TTG ATA TTG TCT ACG A |
| ssmCherryF2/BamHI | ACG GAT CCA TGG GAA AAA TGG CTT CTC TAT TTG CCA CAT TTT TAG TGG TTT TAG T |
| mCherry_CBM22(A)/overR | TCC TTC ATT TTC AAC CAA TCC AAG ACC CAG CTC GTC ATG GGA |
| mCherry_CBM22(A)/overF | TCC CAT GAC GAG CTG GGT CTT GGA TTG GTT GAA AAT GAA GGA |
| CBM22(A)R/SacI | GTG AGC TCT CAG GTT ATG GTT ACA TCG TCA AAA ATG AAA TCT ACA GTG CTG TCG GT |
| ssCBM11_F | GGT TTT AGT GTC ACT TAG CTT AGC AGC TGT CGG TGA AAA AAT G |
| CBM11_ROO/overF | GAT TGG TGC TGG TCT TGG ATT GAT GCA AGC CAC AAA AAT TAT TGA |
| CBM11_ROO/overR | CTT GCA TCA ATC CAA GAC CAG CAC CAA TCA GCT TGA TAT |
| ROO_R/SacI | GTG AGC TCT CAT TGC TCT GCA TAC TCC ACA |
| CaccSP5’ | CACCGGATCCATGGGAAAAATGGCT |
| BIBP3’ | GCCTTTGAAATATTGTTCTCCCAAATCCAATCCAAGACCAGCACCAA |
| BIBPΔ3’ | GCCTACGATATAGATTACTCCCAATACCAATCCAAGACCAGCACCAA |
| QRT-PCR primers |  |
| CIBP-qRT F | AGCATAAACGGTGTGGGAG |
| CIBP-qRT R | CCAAAGTACCGCTCATATCCTG |
| AtACT2-738 F | ACACTGTGCCAATCTACGAGGGTT |
| AtACT2-882 R | ACAATTTCCCGCTCTGCTGTTGTG |
| OseEF-1a-qRT F | TTTCACTCTTGGTGTGAAGCAGAT |
|  | |


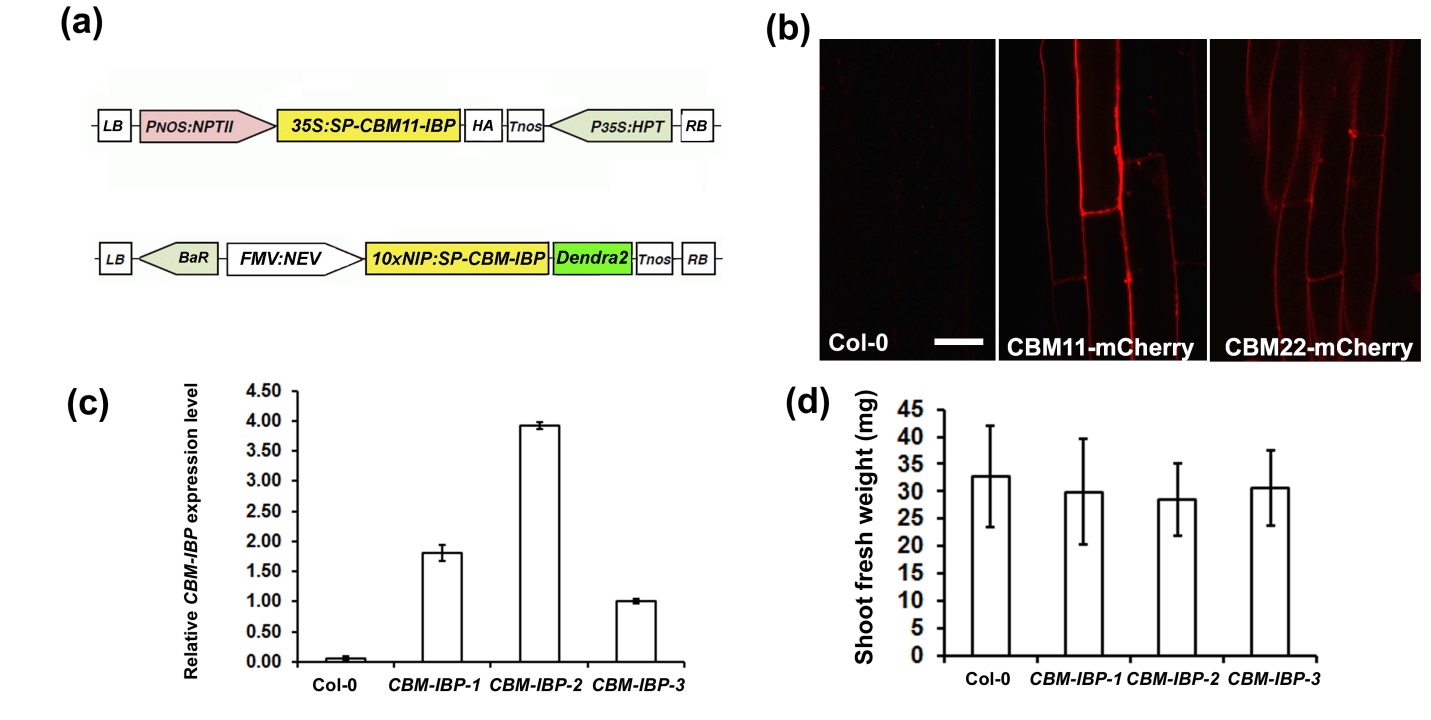


**Supplementary Figure 1**. Expression of *CBM-mCherry* and *CBM-IBP* in Arabidopsis.

(a) Maps of *CBM-IBP* or *IBPΔ* constructs in the pGWB 14 binary vector and *CBM-IBP-Dendra2* in the pIST04 binary vector from the left border (LB) to the right border (RB) of the T-DNA For *pGWB14*-*CBM-IBP/IBP∆*, *CBM-IBP* or *IBP∆* was driven by a 35S promoter, with an N-terminal extensin signal peptide (*SP*) and fused to hemagglutinin (*HA*) tag with a nos terminator (*Tnos*); the selectable marker for plants were kanamycin (*NPTII*) and hygromycin (*HPT*). For *pIST04-CBM-IBP-Dendra2*, *SP-CBM-IBP* was driven by a 10x estrogen inducible promoter *NIP* and fused to *Dendra2* tag with a nos terminator; *FMV:NEV* produces an estrogen binding transcription factor; the selectable marker for plants was BASTA (*BaR*). (b) Localization of CBM11 and CBM22 fused with an extensin signal peptide and a red fluorescent protein (mCherry) showed that CBM11 and 22 can be secreted to cell wall. CBM11-mCherry has stronger signal. (Scale bar = 20 µm.). (c) Relative *CBM-IBP* expression levels in independent homozygous transgenic lines were determined by quantitative real-time PCR analysis using Arabidopsis actin *ACT2* gene as internal reference gene. Values are means ± SD (n = 3 technical replicates). (d) Shoot fresh weight of 18 day old Arabidopsis plants in soil. *CBM-IBP* and Col-0 were comparable, indicating that expression of *CBM-IBP* did not affect growth. Values are means ± SD (*n* = 15 biological replicates).


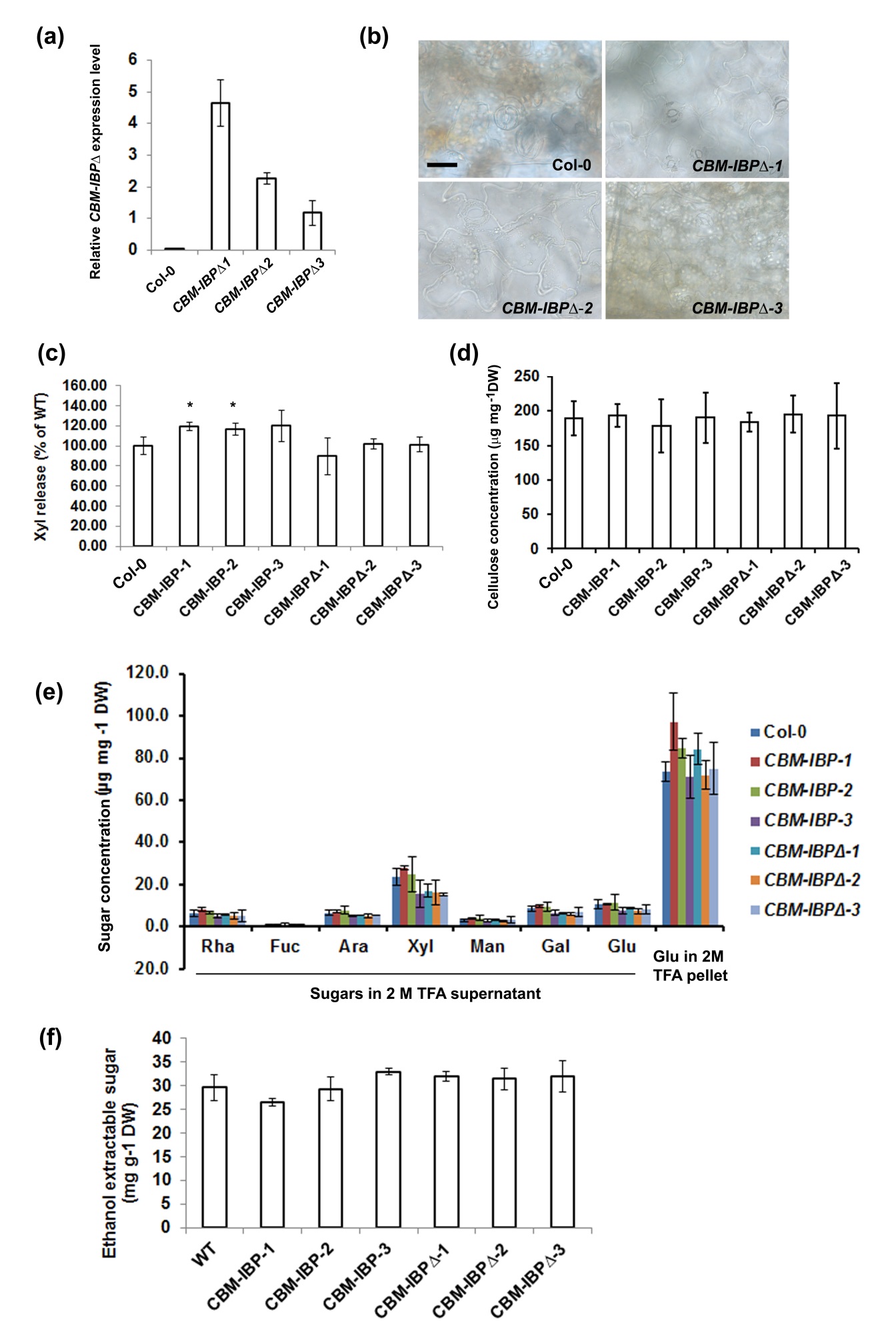


**Supplementary Figure 2**. Cellulose and sugar analysis of Col-0, *CBM-IBP* and *CBM-IBPΔ* biomass.

(a) Relative *CBM-IBP∆* expression levels were determined by quantitative real-time PCR analysis. Values are means ± SD (n = 3 technical replicates). (b) Perls’ Prussian blue staining showed low and similar iron concentration (blue) in Arabidopsis *CBM-IBP∆* and Col-0 leaves. (Scale bar = 50 µm). (c) Xylose (Xyl) yield from *CBM-IBP* Arabidopsis dry shoots was greater than Col-0 and *CBM-IBPΔ.* (d-f) Cellulose, and ethanol insoluble and soluble sugar concentration per dry weight was similar in Col-0, *CBM-IBP* and *CBM-IBPΔ* dry biomass. Rha, rhamose; Fru, fructose; Ara, arabinose; Xyl, xylose; Man, mannose; Gal, galactose; Glu, glucose. Values are means ± SD (*n* = 3 biological replicates).


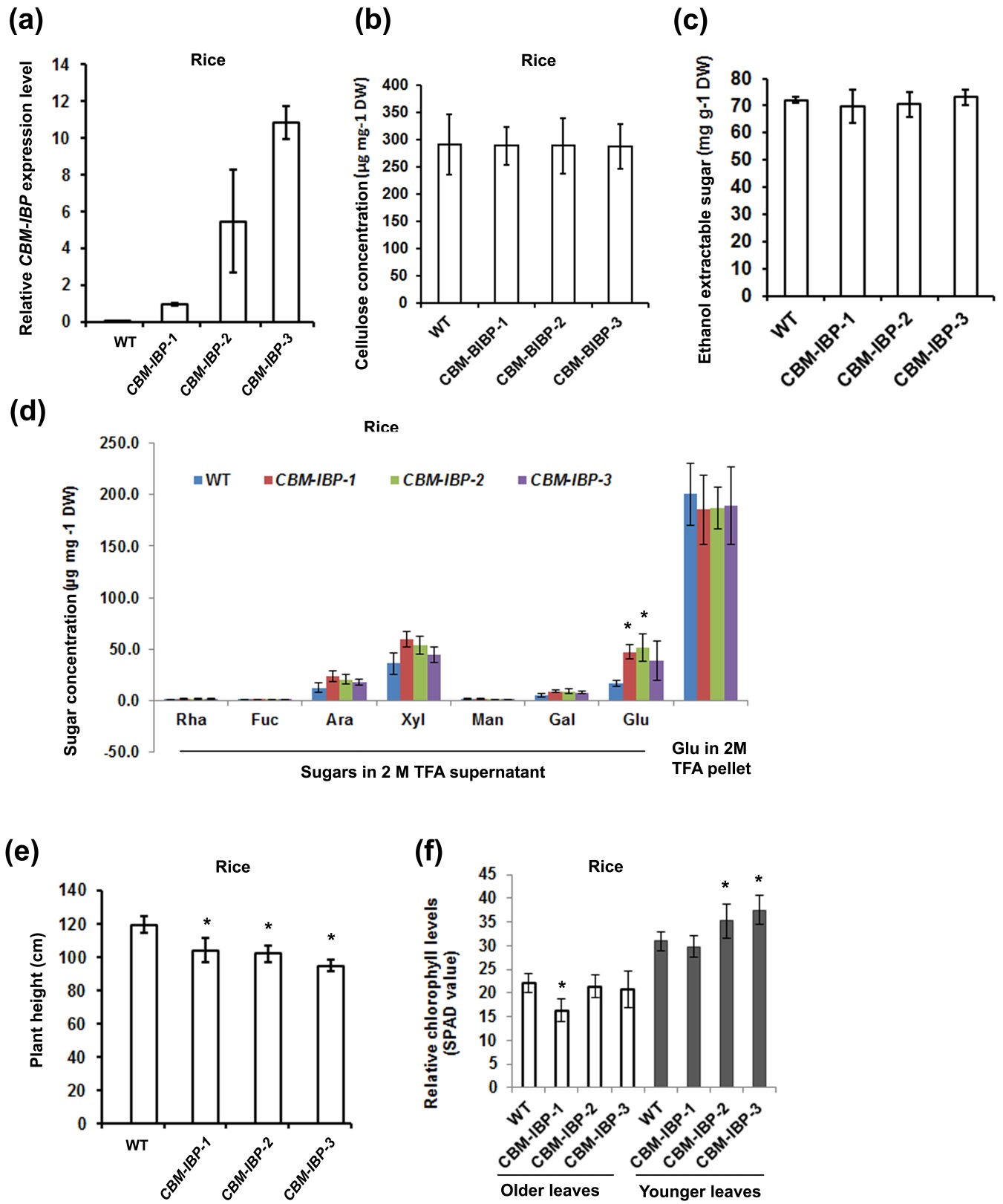


**Supplementary Figure 3**. Characterization of *CBM-IBP* rice plants: expression levels, plant height, chlorophyll concentration, and cellulose and sugar composition. (a) Relative *CBM-IBP* expression levels were determined by quantitative real-time PCR analysis using expression of the rice growth factor gene *OseEF-1α* as an internal reference. Values are means ± SD (n = 3 technical replicates). (b-d) Cellulose, ethanol soluble and insoluble sugar concentration per dry weight was similar in WT and *CBM-IBP* dry biomass. Glucose was higher in the supernatant but lower in the pellet of 2 M trifluoroacetic acid in sugar analysis. Rha, rhamose; Fru, fructose; Ara, arabinose; Xyl, xylose; Man, mannose; Gal, galactose; Glu, glucose. Values are means ± SD (*n* = 3 biological replicates). *, *P* < 0.05, compare to corresponding control. (e) Plant height was measured from shoot-root junction to the tallest flag leaf 120 days after germination. Values are means $\pm$ SD (n = 8 biological replicates). *, *P* < 0.05, compare to corresponding control. (f) Relative chlorophyll levels were determined with SPAD meter in yellow and green flag leaves of 120 days old plants. Values are means $\pm$ SD (n = 12 biological replicates). *, *P* < 0.05, compare to corresponding control.


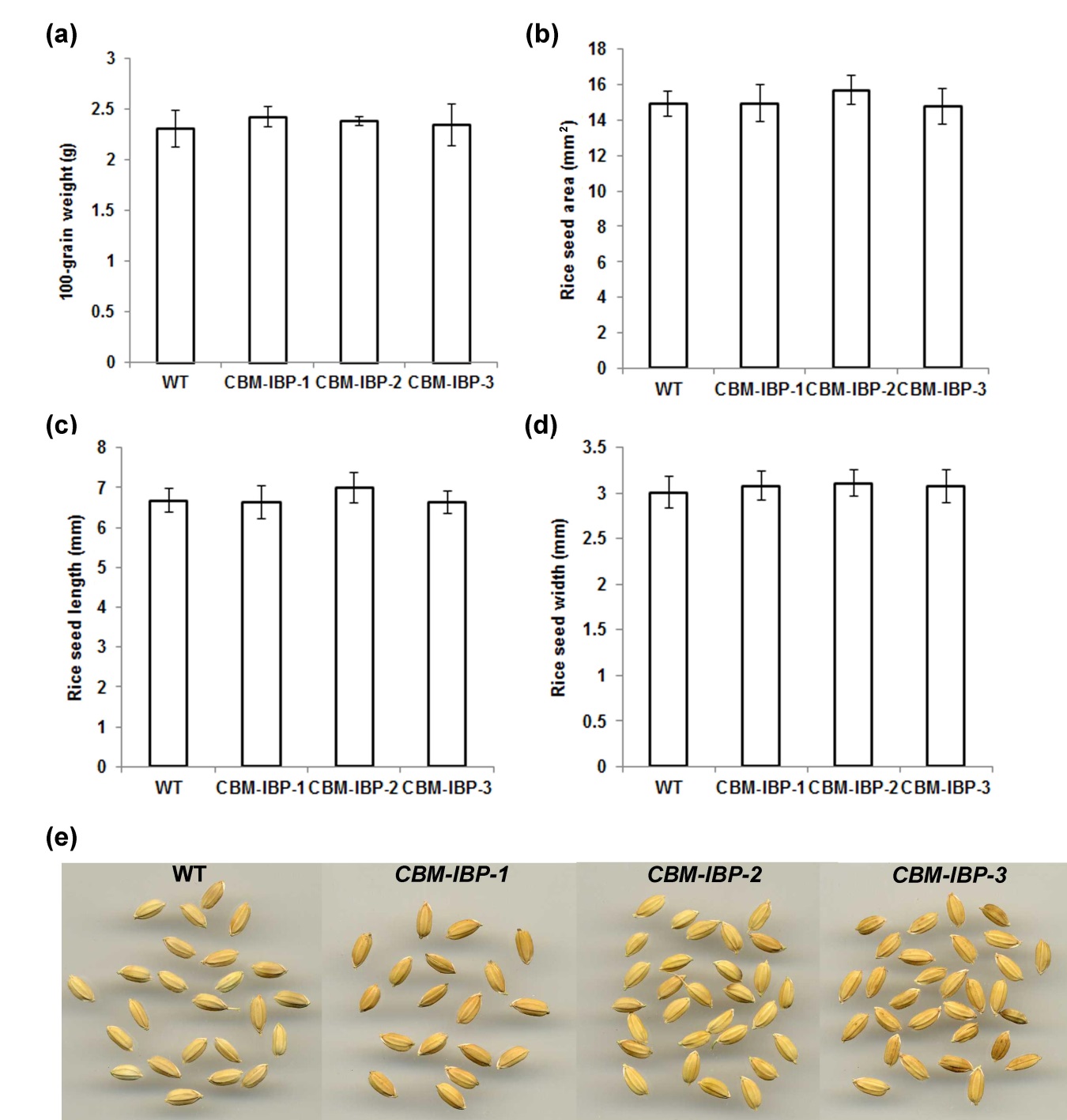


**Supplementary Figure 4.** Characterization of WT and *CBM-IBP* rice grains.

(a) 100-grain weights show that average grain weight of *CBM-IBP* is similar to that of WT. Values are means ± SD (*n* = 6 biological replicates). (b, c, d) Rice seed area, length and width analyzed using SmartGrain software program. (e) Representative image of WT and *CBM-IBP* grains.
